# Anthropometric Measures and Incident Diabetic Nephropathy in Participants With Type 2 Diabetes Mellitus

**DOI:** 10.3389/fendo.2021.706845

**Published:** 2021-08-04

**Authors:** Daniel Nyarko Hukportie, Fu-Rong Li, Rui Zhou, Jia-Zhen Zheng, Xiao-Xiang Wu, Xian-Bo Wu

**Affiliations:** ^1^Department of Epidemiology, School of Public Health, Southern Medical University (Guangdong Provincial Key Laboratory of Tropical Disease Research), Guangzhou, China; ^2^School of Medicine, Southern University of Science and Technology, Shenzhen, China; ^3^Department of General Surgery, 157^th^ Hospital, General Hospital of Guangzhou Military Command, Guangzhou, China

**Keywords:** obesity, waist circumference, waist-to-height ratio, body mass index, diabetic nephropathy

## Abstract

**Background:**

The prevalence of diabetes is on the rise globally coupled with its associated complications, such as diabetic nephropathy (DN). Obesity has been identified as a risk factor for the development of DN but it is still unclear which obesity index is the best predictor of incident DN.

**Methods:**

Data from the participants with type 2 diabetes mellitus (T2DM) in the Action to Control Cardiovascular Risk in Diabetes (ACCORD) study were used to examine the sex-specific association between waist circumference (WC), waist-to-height ratio (WHtR), and body mass index (BMI) with incident DN risk.

**Results:**

Among the 8,887 participants with T2DM (5,489 men and 3,398 women), 5,296 participants (3,345 men and 1,951 women) developed the DN composite outcome during a follow-up period of 24302 person-years. Among men, null associations were observed between all anthropometric measures with incident DN in the multivariate analysis although the 3^rd^ quartile of WHtR showed marginally significant results (*P* = 0.052). However, among women, both central and general obesity measures were associated with increased risks of incident DN. Compared with participants in the WC <88 cm category, the fully adjusted HR and 95% CI for those in the ≥88 cm of WC was 1.35 (95% CI 1.15-1.57). Compared with the lowest quartile, the fully adjusted HRs and 95% CIs for the 2^nd^ to the 4^th^ quartile of WHtR were 1.09 (95% CI 0.96-1.25), 1.12 (95% CI 0.98-1.28), and 1.14 (95% CI 1.00-1.30) respectively; also, compared with the normal BMI category, the fully adjusted HRs and 95% CIs for class I – class III obese were 1.36 (95% CI 1.10 – 1.67), 1.43 (95% CI 1.16 – 1.78) and 1.32 (95% CI 1.05 – 1.66) respectively.

**Conclusions:**

Among participants with T2DM, higher levels of both central and general obesity indexes were associated with DN risk among women but not in men. Women with T2DM should maintain a healthy weight targeted at reducing both central and general obesity to enhance nephroprotection. Trial registration: ClinicalTrials.gov., no. NCT00000620.

## Introduction

Diabetes mellitus, a chronic metabolic disorder, has been recognized as a major global public health burden (1). According to the World Health Organization, an estimated 422 million people were living with diabetes worldwide in 2014, a figure that is projected to rise to about 642 million people by 2040 accompanied by a paralleled increase in its associated complications (2, 3). One of the associated complications of diabetes is diabetic nephropathy (DN) – a microvascular complication characterized by albuminuria and progressive loss of kidney function (4) and is also considered to be the leading cause of end-stage renal disease (ESRD) in the United States (5) and across the globe (6, 7).

Individuals with excess body weight, an indication of excess fat mass may suffer from several cardiometabolic disorders (8). Indeed, a number of studies have reported a positive relationship between renal disease and being overweight among individuals with or without diabetes (9–13). However, some studies have reported conflicting results. For example, in one prospective observational study, the authors found higher levels of body mass index (BMI), a measure of obesity, to be protective against renal function decline in patients with type 2 diabetes mellitus (14). Other studies have also reported U-shaped relationship between obesity measures and microvascular diabetic complications, suggesting that both low and high levels of obesity indexes may act as potential risk factors for renal dysfunction (15, 16).

Body composition varies between men and women, and so is the incidence and health outcomes associated with these sex-specific differences (17). However, most of these studies did not assess the association between obesity indexes and renal outcome stratified by sex. As an indicator of upper body adipose tissue distribution and abdominal obesity, waist circumference (WC) and waist-to-height ratio (WHtR) are considered a potential anthropometric measure of obesity (18). Thus, in the present study, we sought to assess the association between obesity (defined by WC, WHtR, and BMI) and diabetic nephropathy (DN) separately among men and women with T2DM.

In this context, we sought to concurrently investigate the association of WC, WHtR, and BMI with incident DN using data from the Action to Control Cardiovascular Risk in Diabetes (ACCORD) study, which was a randomized, multicenter, double 2 × 2 factorial trial in 10,251 patients with T2DM (18).

## Methods

### Study Participants and Data Collection

ACCORD was a randomized clinical trial of 10,251 participants with type 2 diabetes who were followed with the objective of assessing the health effects of intensive glycemic, lipid, and blood pressure (BP) control as against standard control (19, 20). The design and main results of the ACCORD study have been published previously (20). Briefly, accord had three study arms (1) glycaemia trial (glycated hemoglobin [HbA1c] <6.0% *vs* 7.0% < HbA1c <7.9%); (2) lipid trial (fenofibrate *vs* placebo) and (3) BP trial (systolic BP <120 mmHg *vs* systolic BP <140 mmHg), with all participants involved in the glycaemia trial (21). Recruitment of participants into the study began in January 2001 through to October 2005 from 77 clinical sites across Northern America (i.e. US and Canada) (22). Ethical approval for the ACCORD study was granted by institutional review boards of each clinical site and written informed consent was obtained from all recruited participants (trial registration: ClinicalTrials.gov., no. NCT00000620) (23).

**Figure 1:**
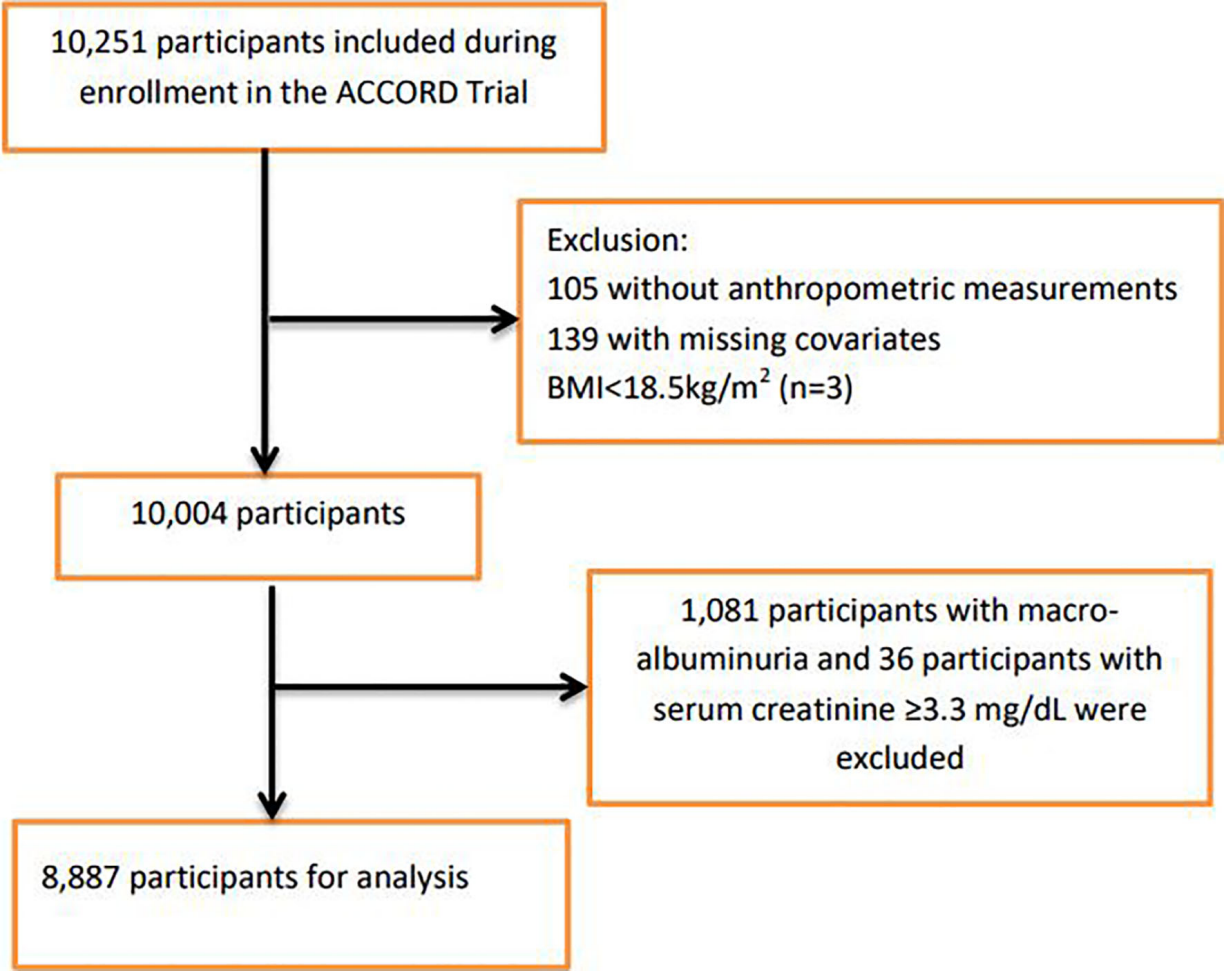
Flowchart for participant enrollment.

For this study, participants were excluded if they had missing data on anthropometric measurements, covariates, or with prevalent baseline nephropathic conditions that formed part of the predefined ACCORD nephropathic incidence event: macro-albuminuria ≥300mg/g and serum creatinine ≥3.3mg/dL (**Supplementary Table 1**). We also excluded three participants that were in the underweight category (BMI <18.5kg/m^2^, n=3). **Figure 1** shows the flowchart of the selection of the analytic sample.

### Exposure Variables

Exposure of interest was anthropometric measurements obtained at baseline, including body mass index (BMI), waist circumference (WC), and waist to height ratio (WHtR). Height was measured using either a steel tape measure marked in centimeters which was hanged vertically on the wall with a headboard at a right angle or commercial stadiometers. Participants were asked to position themselves against the wall having their heels, buttocks, and/or shoulder blades touching the board (tape), and their eyes looking straight, feet together with ankles touching or as close as possible. Bare feet were preferred while light or nylon socks were also allowed during the measurement. The weight of participants was also measured using high-quality scales in clinical practice; during the weighing procedure, participants were asked to wear as little clothing as possible, removing shoes, and other external accessories. Finally, WC was measured at the smallest point between the 10^th^ rib and the iliac crest over bare skin using steel or non-stretching plastic tape. All values were recorded to the nearest decimal place. WHtR and BMI were calculated by dividing WC (cm) by height (cm) and dividing weight (kg) by the height squared (m^2^) respectively. WC was grouped into WHO’s recommended sex-specific groups as follows: men <102 cm and ≥102 cm and women, <88 cm and ≥88 cm (24). WHtR was grouped into sex-specific quartiles as follows: men, Q1 (0.43-0.57), Q2 (0.58-0.61), Q3 (0.62-0.67), and Q4 (0.68-0.86); women, Q1 (0.42-0.59), Q2 (0.60-0.64), Q3 (0.65-0.70), and Q4 (0.71-0.91). BMI was grouped into five categories based on the WHO’s criteria: normal weight (BMI 18.5-24.9 kg/m^2^), overweight (BMI 25.0-29.9 kg/m^2^), class I obese (BMI 30.0-34.9 kg/m^2^), class II obese (BMI 35.0-39.9 kg/m^2^), and class III obese (BMI ≥40.0 kg/m^2^) (24).

### Study Outcome

The study outcome was an incidence of DN defined as (1) doubling of serum creatinine or >20 mL/min decrease in estimated glomerular filtration rate (eGFR); (2) urine albumin/creatinine ratio (UACR) ≥300 mg/g (3) renal failure or ESRD (dialysis) or serum creatinine (SCr) >3.3mg/dL in absence of an acute reversible cause (**Supplementary Table 1**). Participants who experience any one of these events specified by the ACCORD study were considered to have experienced the composite outcome. In the present study, composited DN was used as the primary outcome, while the secondary outcome was the individual outcomes DN mentioned above.

### Statistical Analysis

Participants’ characteristics were described using mean ± standard deviation (SD) or median (25th and 75th percentiles) for continuous variables, depending on whether the data distribution was normal (assessed by the Shapiro-Wilk test); categorical variables were described by frequencies and percentage. Binary logistic regression models were used to examine the relationship between each baseline characteristic and incident DN, where age and sex remained in the models throughout.

Since body composition varies between men and women (17) and our preliminary analysis found interactions between sex and WC and BMI (*P-*interaction = 0.032 and 0.024 respectively) (**Supplementary Table 2**), the analysis was sex-stratified. Cox proportional hazards regression models were used to estimate hazard ratios (HRs) with 95% confidence intervals (CIs) for risk of DN using the lower sex-specific group of WC, lowest sex-specific quartile of WHtR as the reference group as well as the ‘normal’ BMI category as the reference group. The participants were followed from baseline until the time of development of the DN outcome, time of death, loss to follow-up, or the end of the follow-up, whichever occurred first.

Baseline covariates with *P <*0.1 from the univariate analysis were subsequently included stepwise logistic regression analysis to identify the most relevant variables contributing to risk of DN. All covariates were treated as time-dependent covariates. Tests for linear trends were carried out by entering the median value of each category of WC, WHtR, and BMI as a continuous variable in the models.

Further exploration was carried out to examine possible interactions between each SD increase in WC, WHtR, BMI, and the following baseline characteristics of the participants with respect to the primary outcome: age (< 65 or ≥65 years), diabetes duration (<9 or ≥ 9 years), BP *vs* Lipid (BP treatment arm or lipid treatment arm), and SBP (< 140 or ≥140 mmHg). The statistical significance of the interactions was assessed by adding a multiplicative term to the Cox models. Sensitivity analyses were also performed by excluding participants with any serious adverse event (attributed to study medication and not related to hypoglycemia) that occurred in the first 2 years of follow-up.

All statistical analyses were 2-sided, and we considered a *p-*value of < 0.05 to be statistically significant. All analyses were performed using Stata (version 16 MP; StataCorp, College Station, Texas).

## Results

Among the 8,887 participants with T2DM, 5,489 (61.8%) were men and 3,398 (38.2%) were women. **Table 1** summarizes the baseline characteristics stratified by subjects who subsequently developed DN and those who did not. Compared with those without DN, those who developed DN were more likely to be men, white, belonged to the lipid treatment arm of ACCORD, had higher levels of WC, WHtR, BMI, HbA1c, SBP, UACR, but lower HDL, LDL, and serum creatinine levels. We also presented the baseline characteristics of the study participants by quartiles of WC, WHtR, BMI categories (**Supplementary Tables 3** and **5**). Patients with a larger WC, WHtR, and BMI were more likely to be younger, white, and have higher diastolic BP. Higher WHtR and BMI were more likely to be associated with being female, whereas higher levels of WC were associated with being a male.

**Table 1:** Baseline characteristics stratified by subjects who subsequently developed DN and those who did not, after adjusting for age and sex.

Characteristics	Total (n=8887)	No DN (n=3591)	DN cases (n=5296)	*P*-value*
Age, yrs	62.0 (57.7, 67.1)	62.2 (57.7, 67.6)	61.9 (57.7, 66.9)	0.024
Gender				
Men	5489 (61.8)	2144 (59.7)	3345 (63.2)	0.001
Race				
White	5596 (63.0)	2208 (61.5)	3388 (64.0)	0.029
Education				
College graduate or more	2333 (26.3)	984 (27.4)	1349 (25.5)	0.011
WC, cm	106.7 (97, 116)	105.5 (96.5, 115)	106.7 (97.8, 116.8)	<0.001
Weight, kg	92.6 (80.7, 105.7)	91.9 (79.8, 105.5)	93.2 (81.1, 105.9)	0.125
WHtR	0.62 (0.57, 0.68)	0.62 (0.57, 0.68)	0.63 (0.57, 0.68)	<0.001
BMI, kg/m2	31.8 (28.2, 35.8)	31.5 (28.1, 35.7)	32.0 (28.2, 36.0)	0.004
HbA1c, %	8.1 (7.6, 8.8)	8.0 (7.5, 8.7)	8.2 (7.6, 8.9)	<0.001
Duration of diabetes, yrs	9 (5, 15)	9 (5, 15)	9 (5, 15)	0.009
Glycemia arm				
Intensive	4449 (50.1)	1818 (50.6)	2631 (49.7)	0.382
BP vs Lipid				
Lipid treatment	4743 (53.4)	1719 (47.9)	3024 (57.1)	<0.001
Insulin use				
Yes	2994 (33.7)	1186 (33.0)	1808 (34.1)	0.187
Cigarette smoking				
Yes	1219 (13.7)	484 (13.5)	735 (13.9)	0.940
SBP, mmHg	135 (124, 146)	133 (123, 144)	136 (125, 147)	<0.001
DBP, mmHg	75 (68, 82)	75 (68, 81)	75 (68, 82)	0.310
CVD				
Yes	3087 (34.7)	1170 (32.6)	1917 (36.2)	0.003
Dyslipidemia				
Yes	6968 (78.4)	2841 (79.1)	4127 (77.9)	0.153
HDL, mg/dL	40 (34, 48)	41 (35, 49)	39 (34, 47)	<0.001
LDL, mg/dL	101 (81, 125)	102 (82, 126)	100 (80, 124)	0.012
eGFR, ml/min/1.73m2	89.8 (76.3, 105.2)	80.5 (69, 92.3)	92.2 (79.7, 109)	<0.001
UACR, mg/g	13 (7, 34)	10 (6, 23)	15 (7, 44)	<0.001
Serum creatinine, mg/dL	0.9 (0.8, 1)	0.9 (0.8, 1.1)	0.8 (0.7, 1)	<0.001

Data are shown as medians (interquartile range) for continuous variables and as frequencies and percentage for categorical variables.

WC, Waist circumference; WHtR, Waist-to-height ratio; BMI, Body mass index; SBP, blood pressure; DBP, blood pressure; CVD, Cardiovascular disease; HDL, high density lipoprotein; LDL, Low density lipoprotein; eGFR, Estimated glomerular filtration rate; UACR, Urinary albumin creatinine ratio.

*P values refer to results after analyses with adjustment for age and sex.

Over 24,302 person-years of follow-up, 5,296 participants (3,345 men and 1,951 women) developed the DN composite outcome (2.3 per 10 person-years in men; 2.1 per 10 person-years in women). Among men, higher levels of WC, WHtR, and BMI in the unadjusted models were associated with an increased risk of incident DN, but all lost significance in the full models although the 3^rd^ quartile of WHtR showed marginally significant results (*P* = 0.052). Compared with the lowest quartile, the fully adjusted hazard ratio (HR) and 95% confidence interval (CI) for the 3rd quartile of WHtR was 1.10 (95% CI 1.00-1.21) among men (**Table 2**). On the contrary, among women, higher levels of WC, WHtR, and BMI were all associated with incident DN in the full models. The fully-adjusted HR (95% CIs) for WC among women was 1.35 (1.15-1.57) for the highest group (≥ 88cm), compared with the lower group (*P*-trend <0.001); the fully-adjusted HR (95% CIs) for WHtR among women were 1.09 (0.96-1.25), 1.12 (0.98-1.28), and 1.14 (1.00-1.30) for the 2^nd^ to the 4^th^ quartile compared with the 1st, respectively (*P*-trend = 0.047); also, the fully-adjusted HR (95% CIs) for BMI among women were 1.28 (1.04-1.58), 1.36 (1.10 – 1.67), 1.43 (1.16-1.78) and 1.32 (1.05 – 1.66) for the overweight category to Class III obese category, compared with the normal weight category, respectively (*P*-trend = 0.035) (**Table 3**). Similar results were obtained in examining the association between each 1 SD increase in WC, WHtR, and BMI with incident DN among men and women. The results for men showed no significant association between each SD increase in WC, WHtR, and BMI with incident DN whereas among women, each SD increase in WC, WHtR, and BMI were associated with a significantly increased risk of DN (**Tables 2** and **3**).

**Table 2:** Hazard ratios for incident DN, by WC, WHtR, and BMI among men with diabetes (n=5,489).

	Events	Incidence Density (Per 10 000 Person Days)	Model 1	Model 2
**WC group**				
<102cm	971	5.7	Ref.	Ref.
≥102cm	2374	6.4	1.11 (1.03-1.19)*	1.03 (0.95-1.11)
*P* for trend			0.007	0.497
				
Each SD increase in WC			1.06 (1.02-1.09)**	1.01 (0.97-1.05)
				
**WHtR Quartiles**				
Q1	790	5.4	Ref.	Ref.
Q2	814	6.1	1.09 (0.98-1.20)	1.07 (0.97-1.18)
Q3	874	6.4	1.16 (1.06-1.28)**	1.10 (1.00-1.21)**^∞^**
Q4	867	6.8	1.20 (1.08-1.31)***	1.06 (0.96-1.17)
*P* for trend			0.000	0.230
				
Each SD increase in WHtR			1.07 (1.04-1.11)***	1.02 (0.98-1.05)
				
**BMI categories**				
Normal	271	5.7	Ref.	Ref.
Overweight	1073	5.9	1.04 (0.91-1.19)	1.04 (0.91-1.19)
Class I obese	1164	6.3	1.09 (0.96-1.25)	1.08 (0.94-1.24)
Class II obese	597	6.1	1.06 (0.92-1.22)	0.97 (0.84-1.12)
Class III obese	240	7.5	1.25 (1.05-1.48)*	1.13 (0.95-1.35)
*P* for trend			0.031	0.767
Each SD increase in BMI			1.04 (1.01- 1.08)*	1.01 (0.97-1.04)

WC, Waist circumference; WHtR, Waist to height ratio; BMI, Body mass index.

Model 1, unadjusted.

Model 2 adjusted for baseline age(cont.), race (white or non-white), BP vs lipid (BP treatment and lipid treatment), duration of diabetes (cont.) systolic BP (cont.), HbA1c (cont.), eGFR (cont.), UACR (cont.), CVD history (yes or no), high-density lipoprotein (cont.) and low-density lipoprotein (cont.).

P-value notation: *P < 0.05. **P < 0.01. ***P < 0.001.

^**∞**^P value = 0.052.

**Table 3:** Hazard ratios for incident DN, by WC, WHtR, and BMI among women with diabetes (n=3,398).

	Events	Incidence Density (Per 10 000 Person Days)	Model 1	Model 2
**WC group**				
<88cm	203	4.6	Ref.	Ref.
≥88cm	1748	5.8	1.21 (1.05-1.40)*	1.35 (1.15-1.57)***
*P* for trend			0.009	0.000
				
Each SD increase in WC			1.06 (1.01-1.10)*	1.07 (1.02-1.12)*
				
**WHtR Quartiles**				
Q1	466	5.2	Ref.	Ref.
Q2	480	5.5	1.04 (0.92-1.19)	1.09 (0.96-1.25)
Q3	484	5.7	1.08 (0.95-1.23)	1.12 (0.98-1.28)
Q4	521	6.3	1.17 (1.04-1.33)*	1.14 (1.00-1.30)*
*P* for trend			0.010	0.047
				
Each SD increase in WHtR			1.06 (1.02-1.11)**	1.07 (1.02-1.12)**
				
**BMI categories**				
Normal	130	4.8	Ref.	Ref.
Overweight	455	5.4	1.09 (0.90-1.33)	1.28 (1.04-1.58)*
Class I obese	608	5.6	1.12 (0.93-1.35)	1.36 (1.10-1.67)**
Class II obese	477	6.3	1.24 (1.02-1.51)*	1.43 (1.16-1.78)**
Class III obese	281	5.6	1.14 (0.93-1.41)	1.32 (1.05-1.66)*
*P* for trend			0.065	0.035
				
Each SD increase in BMI			1.04 (1.00 - 1.09)	1.06 (1.01-1.11)*

WC, Waist circumference; WHtR, Waist to height ratio; BMI, Body mass index.

Model 1, unadjusted.

Model 2 adjusted for baseline age(cont.), race (white or non-white), BP vs lipid (BP treatment and lipid treatment), duration of diabetes (cont.) systolic BP (cont.), HbA1c (cont.), eGFR (cont.), UACR (cont.), CVD history (yes or no), high-density lipoprotein (cont.) and low-density lipoprotein.

P-value notation: *P < 0.05. **P < 0.01. ***P < 0.001.

Analysis of each DN diagnostic event that comprised the composite outcome [(1) doubling of SCr or >20 mL/min/1.73m^2^ decrease in eGFR; (2) macroalbuminuria; and (3) renal failure or ESRD (dialysis) or SCr >3.3mg/dL in absence of an acute reversible cause] showed that among men, WC ≥ 102 cm was associated with reduced risk of macroalbuminuria (**Supplementary Table 7**). All other analyses for the relationship of WC, WHtR, and BMI with other DN diagnostic outcomes were null for the full models among men (**Supplementary Tables 6–8**). Among women, however, higher levels of WC, WHtR, and BMI were all associated with an increased risk of doubling of SCr or > 20 mL/min/1.73m^2^ decrease in eGFR (**Supplementary Tables 9**). The results for the relationship between WC, WHtR, and BMI with other DN outcomes were null for women (**Supplementary Tables 10** and **11**)

Subgroup analyses of the HRs of DN for each SD increase in the WC, WHtR, and BMI are shown in **Supplementary Tables 12–14**. Generally, there were no significant interactions between various subgroups among men. However, among women, we found that allocation to BP/lipid treatment group significantly modified the relation between each 1SD increase in the WC, WHtR and BMI and the risk of incident DN (*P*_interaction_ = 0.044, 0.028 and 0.026 respectively). The links between WC, WHtR and BMI, and incident DN were stronger among participants assigned to the BP treatment group than those assigned to the lipid treatment group (**Supplementary Tables 12–14**). Also, we found significant modified effect between duration of diabetes and each 1SD increase in BMI among women (*P*_interaction_ = 0.023). The link between BMI and incident DN was stronger among participants with < 9years of diabetes duration than in those in ≥ 9years (**Supplementary Table 14**). The results were not materially changed after excluding those who had serious adverse events in the first 2 years of follow-up except for the quartiles of WHtR which lost significance among women, whereas the 3rd quartile of WHtR became fully significant among men (**Supplementary Tables 15** and **16**).

## Discussion

In this *post-hoc* study, using data from 8,887 patients with T2DM, we examined the association of anthropometric measures with the risk of DN. The results from the present study suggest that obesity, as defined by WC, WHtR, and BMI, may be associated with an increased risk of DN in women but not in men after adjustment for potential-related confounders.

Mixed results have been reported by previous studies assessing the association between central obesity and general obesity with renal diseases in participants with or without T2DM. The results of the present study are consistent with some studies that found detrimental associations between obesity indices and renal function in women but not in men. In a study to assess the sex difference in the association between anthropometric measures and albuminuria in a sample of 3,841Korean adults, the researchers found both WC and BMI were associated with albuminuria in women but not men (25). Lin et al. in a study of 2,350 middle-aged Chinese participants also found central obesity indicators to be associated with the development of albuminuria in women but not men (26). A similar finding was observed in a sample of 569 T2DM patients (27). However, these studies are either cross-sectional studies or smaller sample sizes.

Using a sample of nearly 9,000 participants with T2DM from a randomized clinical trial, we provided important data supporting evidence of higher levels of WC, WHtR, and BMI as a risk factor for DN in women. The pathological mechanisms underlying these associations of obesity and renal function decline are not entirely clear. However, adipose tissues have been implicated in the release of inflammatory mediators such as interleukin-6 and tumor necrosis factor α, and also linked to the cause of insulin resistance (28). The combination of these factors may lead to structural changes in the kidneys and eventually result in obesity-related glomerulopathy (29). Furthermore, excess visceral adipose tissue may also provoke hemodynamic changes in the kidneys which subsequently cause elevated glomerular capillary pressure leading to possible renal damage (28).

It must be noted that other studies found obesity markers to be associated with increased risk of renal function decline in men but not in women (30–33). The reason for the insignificant relationship between anthropometric measures and the renal outcomes in men with T2DM in our study is unclear. This may suggest that some other factors such as sex hormones may play important role in the pathological mechanism between men and women. Another possibility is that men generally have higher levels of lean body mass which have been associated with better health outcomes (34, 35); and may account for the null associations observed in the relationship between the obesity indexes and incident DN among men (25, 36). Further studies will be necessary to elucidate the sex-specific association of anthropometric measures in women with T2DM and the lack thereof among men.

Our findings suggest that both central and general obesity indicators may be an important measure to assess among women with T2DM. These central adiposity indexes like the general obesity indices, not only come at relatively low cost, easy to assess, and easily carried out in any clinical setting, but have an added advantage of having a high correlation with visceral adiposity than the general obesity indicator of BMI (37). Clinicians might want to consider efforts to increase awareness and prevention of both central and general obesity to reduce DN risk as the prevalence of diabetes is projected to increase globally (37).

The strengths of our study include the relatively large sample size, comprehensive clinical and biological characterization of participants, and prospective data collection. There was also a special nephropathic examination at baseline and at subsequent visits, which permitted the accurate assessment of the effect of anthropometric measures on the risk of DN. The attrition of participants in this study was also very minimal. However, our study has some limitations. Firstly, our study lacks the ability to determine causality because of its observational study design. Secondly, due to the limited number of participants in the underweight category in this sample, we could not establish any relationship for individuals who are underweight. Thirdly, the generalization of these findings to other populations may be limited since all study participants were from northern America and there may be variability in body composition across different populations.

In conclusion, among participants with T2DM, higher levels of both central and general obesity measures were associated with DN risk among women but not in men. Women with T2DM should maintain a healthy weight targeted at reducing both abdominal fat and general obesity to enhance nephroprotection.

## Data Availability Statement

Publicly available datasets were analyzed in this study. This data can be found here: https://biolincc.nhlbi.nih.gov/studies/accord/(Repository: Biologic Specimen and Data Repository).

## Ethics Statement

The studies involving human participants were reviewed and approved by ACCORD Protocol Review Committee appointed by National Heart, Lung, and Blood Institute (NHLBI) & Local institutional review boards of participating clinical sites. The patients/participants provided their written informed consent to participate in this study.

## Author Contributions

DH and F-RL conceived, analyzed the data and wrote the manuscript. RZ, J-ZZ, X-XW, and X-BW contributed to the discussion and reviewed/edited the manuscript. All authors contributed to the article and approved the submitted version.

## Funding

This study was supported by Open Project of Guangdong Provincial Key Laboratory of Tropical Disease Research and Guangdong Basic and Applied Basic Research Foundation.

## Conflict of Interest

The authors declare that the research was conducted in the absence of any commercial or financial relationships that could be construed as a potential conflict of interest.

## Publisher’s Note

All claims expressed in this article are solely those of the authors and do not necessarily represent those of their affiliated organizations, or those of the publisher, the editors and the reviewers. Any product that may be evaluated in this article, or claim that may be made by its manufacturer, is not guaranteed or endorsed by the publisher.
