# Supplementary material for: Anthropometric Measures and Incident Diabetic Nephropathy in Participants With Type 2 Diabetes Mellitus
Source: Front Endocrinol (Lausanne). 2021 Aug 4;12:706845. doi: 10.3389/fendo.2021.706845 (PMC8371436; doi:10.3389/fendo.2021.706845)
Supplement: Supplementary file 1 [file Table_1.docx]

**Supplementary Material**

**Supplementary Table 1** Definition of DN and their frequency of assessment

| **Outcome** | **Definition** | **Assessment Frequency** |
| --- | --- | --- |
| Nephro-1 | SCr doubling or >20 mL/min/1.73 m^2^ decrease in eGFR | Every 4 month |
| Nephro-2 | Development of macro-albuminuria (uacr>=300mg/g) | Annually |
| Nephro-3 | Renal failure OR ESRD (dialysis) OR SCr>3.3 mg/dL in absence of an acute reversible cause | Every 4 month |

Abbreviations: SCr = Serum creatinine. eGFR = Estimated glomerular filtration rate. uacr = Urinary albumin/creatinine ratio. ESRD = End stage renal disease

**Supplementary Table 2** Analysis for interaction of Sex with WC, WHtR and BMI on risk of incident DN

| **Factor** | **Events/n** | **HR (95% CI)** | ***p* for interaction** |
| --- | --- | --- | --- |
| **WC, cm** |  |  |  |
| Male | 3,344/ 5,488 | 1.01 (0.97 - 1.05) | 0.032 |
| Female | 1,951/ 3,397 | 1.07 (1.02 - 1.12) * |  |
| **WHtR** |  |  |  |
| Male | 3,344/ 5,488 | 1.02 (0.98 – 1.06) | 0.082 |
| Female | 1,951/ 3,397 | 1.07 (1.02 - 1.11) ** |  |
| **BMI, kg/m^2^** |  |  |  |
| Male | 3,344/ 5,488 | 1.01 (0.97 – 1.05) | 0.024 |
| Female | 1,951/ 3,397 | 1.05 (1.01 - 1.10) * |  |
| Each stratification was adjusted for baseline age, race, BP vs lipid (BP treatment and lipid treatment), duration of diabetes systolic BP, HbA1c, eGFR, UACR, CVD history, high-density lipoprotein and low-density lipoprotein except for the stratification factor itself. Note: CI = confidence interval, HR = hazard ratio, WC = waist circumference WHtR = waist-to-height ratio, BMI = body mass index *p*-value notation: *: p<0.05. **: p<0.01. ***: p<0.001 | | | |

**Supplementary Table 3** Characteristics of study participants according to quartiles of WC

| Characteristics | Total (n=8887) | Q1 (n=2249) | Q2 (n=2469) | Q3 (n=1956) | Q4 (n=2213) | p-value |
| --- | --- | --- | --- | --- | --- | --- |
| Age, yrs | 62 (57.7, 67.1) | 62.4 (57.9, 68) | 62.4 (57.9, 67.7) | 62 (57.8, 67.1) | 61.3 (57.4, 65.6) | <0.001 ^Ψ^ |
| Gender |  |  |  |  |  | <0.001 |
| Women | 3398 (38.2%) | 1109 (49.3%) | 893 (36.2%) | 692 (35.4%) | 704 (31.8%) |  |
| Men | 5489 (61.8%) | 1140 (50.7%) | 1576 (63.8%) | 1264 (64.6%) | 1509 (68.2%) |  |
| Race |  |  |  |  |  | <0.001 |
| White | 5596 (63.0%) | 1040 (46.2%) | 1578 (63.9%) | 1332 (68.1%) | 1646 (74.4%) |  |
| Non-white | 3291 (37.0%) | 1209 (53.8%) | 891 (36.1%) | 624 (31.9%) | 567 (25.6%) |  |
| Education |  |  |  |  |  | <0.001 |
| Less than high school graduate | 1295 (14.6%) | 361 (16.1%) | 351 (14.2%) | 294 (15.0%) | 289 (13.1%) |  |
| High school grad (or GED) | 2342 (26.4%) | 613 (27.3%) | 648 (26.3%) | 512 (26.2%) | 569 (25.7%) |  |
| Some college or technical school | 2912 (32.8%) | 641 (28.5%) | 794 (32.2%) | 657 (33.6%) | 820 (37.1%) |  |
| College graduate or more | 2333 (26.3%) | 632 (28.1%) | 675 (27.4%) | 492 (25.2%) | 534 (24.1%) |  |
| WC, cm | 106.7 (97, 116) | 91.4 (86.4, 94.4) | 102 (100, 105) | 111.8 (109.2, 113.5) | 123 (119.4, 128.5) | <0.001 ^Ψ^ |
| Weight, kg | 92.6 (80.7, 105.7) | 74.5 (66.7, 81.2) | 88.5 (82.1, 95.3) | 98.9 (92.1, 106.2) | 113.4 (104.2, 122.5) | <0.001 ^Ψ^ |
| WHtR | .6 (.6, .7) | .5 (.5, .6) | .6 (.6, .6) | .6 (.6, .7) | .7 (.7, .8) | <0.001 ^Ψ^ |
| BMI, kg/m^2^ | 31.8 (28.2, 35.8) | 26.6 (24.6, 28.9) | 30.3 (28.3, 32.6) | 33.3 (31.2, 35.7) | 37.8 (35.1, 40.9) | <0.001 ^Ψ^ |
| Hba1c, % | 8.1 (7.6, 8.8) | 8 (7.5, 8.9) | 8.1 (7.6, 8.8) | 8.1 (7.6, 8.8) | 8.1 (7.6, 8.9) | 0.060 ^Ψ^ |
| Duration of diabetes, yrs | 9 (5, 15) | 10 (5, 15) | 10 (5, 15) | 9 (5, 14) | 9 (5, 14) | <0.001 ^Ψ^ |
| Glycemia |  |  |  |  |  | 0.99 |
| Standard | 4438 (49.9%) | 1119 (49.8%) | 1235 (50.0%) | 983 (50.3%) | 1101 (49.8%) |  |
| Intensive | 4449 (50.1%) | 1130 (50.2%) | 1234 (50.0%) | 973 (49.7%) | 1112 (50.2%) |  |
| BP vs Lipid |  |  |  |  |  | <0.001 |
| BP treatment | 4144 (46.6%) | 1152 (51.2%) | 1195 (48.4%) | 834 (42.6%) | 963 (43.5%) |  |
| Lipid treatment | 4743 (53.4%) | 1097 (48.8%) | 1274 (51.6%) | 1122 (57.4%) | 1250 (56.5%) |  |
| Cigarette smoking |  |  |  |  |  | <0.001 |
| Yes | 1219 (13.7%) | 379 (16.9%) | 318 (12.9%) | 254 (13.0%) | 268 (12.1%) |  |
| No | 7668 (86.3%) | 1870 (83.1%) | 2151 (87.1%) | 1702 (87.0%) | 1945 (87.9%) |  |
| Systolic BP, mmHg | 135 (124, 146) | 136 (125, 147) | 135 (125, 146) | 134 (124, 147) | 134 (123, 145) | 0.007 |
| Diastolic BP, mmHg | 75 (68, 82) | 74 (67, 81) | 74 (67, 81) | 75 (68, 83) | 76 (68, 82) | <0.001 |
| CVD |  |  |  |  |  | 0.014 |
| Yes | 3087 (34.7%) | 723 (32.1%) | 855 (34.6%) | 711 (36.3%) | 798 (36.1%) |  |
| No | 5800 (65.3%) | 1526 (67.9%) | 1614 (65.4%) | 1245 (63.7%) | 1415 (63.9%) |  |
| Dyslipidemia |  |  |  |  |  | 0.068 |
| Yes | 1919 (21.6%) | 479 (21.3%) | 506 (20.5%) | 413 (21.1%) | 521 (23.5%) |  |
| No | 6968 (78.4%) | 1770 (78.7%) | 1963 (79.5%) | 1543 (78.9%) | 1692 (76.5%) |  |
| HDL, mg/dL | 40 (34, 48) | 44 (36, 52) | 40 (34, 48) | 39 (34, 46) | 38 (32, 45) | <0.001 ^Ψ^ |
| LDL, mg/dL | 101 (81, 125) | 104 (84, 129) | 101 (82, 125) | 99 (80, 124) | 98 (79, 121) | <0.001 ^Ψ^ |
| eGFR, ml/min/1.73m^2^ | 89.8 (76.3, 105.2) | 89.7 (76.5, 106) | 89.2 (75.8, 104.7) | 89.9 (76.3, 105.1) | 90.2 (76.5, 105.2) | 0.17 ^Ψ^ |
| UACR, mg/g | 13 (7, 34) | 11 (7, 30) | 12 (7, 32) | 13 (7, 32) | 15 (7, 44) | <0.001 ^Ψ^ |
| Serum creatinine, mg/dL | .9 (.8, 1) | .9 (.7, 1) | .9 (.8, 1) | .9 (.8, 1) | .9 (.8, 1) | <0.001 ^Ψ^ |
| Abbreviations: WC= Waist circumference, WHtR= Waist-to-height ratio, BMI= Body mass index, BP= blood pressure, CVD= Cardiovascular disease, HDL= high density lipoprotein, LDL= Low density lipoprotein, eGFR= Estimated glomerular filtration rate, UACR= Urinary albumin creatinine ratio  Ψ: P-values from the rank sum test. All other p-values are from the Pearson chi-square test. | | | | | | |

**Supplementary Table 4** Characteristics of study participants according to quartiles of WHtR

| Characteristics | Total (n=8887) | Q1 (n=2224) | Q2 (n=2220) | Q3 (n=2224) | Q4 (n=2219) | p-value |
| --- | --- | --- | --- | --- | --- | --- |
| Age, yrs | 62 (57.7, 67.1) | 62.15 (57.6, 67.9) | 62.5 (57.9, 67.75) | 62.05 (57.8, 67.2) | 61.4 (57.4, 65.8) | <0.001 ^Ψ^ |
| Gender |  |  |  |  |  | <0.001 |
| Women | 3398 (38.2%) | 662 (29.8%) | 711 (32.0%) | 888 (39.9%) | 1137 (51.2%) |  |
| Men | 5489 (61.8%) | 1562 (70.2%) | 1509 (68.0%) | 1336 (60.1%) | 1082 (48.8%) |  |
| Race |  |  |  |  |  | <0.001 |
| White | 5596 (63.0%) | 1194 (53.7%) | 1380 (62.2%) | 1451 (65.2%) | 1571 (70.8%) |  |
| Non-white | 3291 (37.0%) | 1030 (46.3%) | 840 (37.8%) | 773 (34.8%) | 648 (29.2%) |  |
| Education |  |  |  |  |  | <0.001 |
| Less than high school graduate | 1295 (14.6%) | 284 (12.8%) | 311 (14.0%) | 365 (16.4%) | 335 (15.1%) |  |
| High school grad (or GED) | 2342 (26.4%) | 558 (25.1%) | 594 (26.8%) | 595 (26.8%) | 595 (26.8%) |  |
| Some college or technical school | 2912 (32.8%) | 671 (30.2%) | 714 (32.2%) | 743 (33.4%) | 784 (35.4%) |  |
| College graduate or more | 2333 (26.3%) | 710 (31.9%) | 600 (27.0%) | 520 (23.4%) | 503 (22.7%) |  |
| WC, cm | 106.7 (97, 116) | 91.4 (86.4, 96.5) | 102.5 (97.8, 106.7) | 110.5 (105.5, 115.1) | 121.9 (116.8, 128.3) | <0.001 ^Ψ^ |
| Weight, kg | 92.6 (80.7, 105.7) | 78.5 (69.4, 88) | 89.4 (80, 99.2) | 96.6 (86.95, 108) | 108 (96.6, 120.2) | <0.001 ^Ψ^ |
| WHtR | 0.6 (0.6, 0.7) | 0.5 (0.5, 0.6) | 0.6 (0.6, 0.6) | 0.6 (0.6, 0.7) | 0.7 (0.7, .8) | <0.001 ^Ψ^ |
| BMI, kg/m^2^ | 31.8 (28.2, 35.8) | 26.6 (24.6, 28.3) | 30.1 (28.4, 32.1) | 33.5 (31.4, 35.4) | 38.3 (35.8, 41.2) | <0.001 ^Ψ^ |
| Hba1c, % | 8.1 (7.6, 8.8) | 8 (7.5, 8.8) | 8 (7.6, 8.8) | 8.1 (7.6, 8.8) | 8.2 (7.6, 8.9) | 0.001 ^Ψ^ |
| Duration of diabetes, yrs | 9 (5, 15) | 10 (5, 15) | 9 (5, 15) | 9 (5, 14) | 9 (5, 15) | 0.002 ^Ψ^ |
| Glycemia |  |  |  |  |  | 0.78 |
| Standard | 4438 (49.9%) | 1095 (49.2%) | 1113 (50.1%) | 1128 (50.7%) | 1102 (49.7%) |  |
| Intensive | 4449 (50.1%) | 1129 (50.8%) | 1107 (49.9%) | 1096 (49.3%) | 1117 (50.3%) |  |
| BP vs Lipid |  |  |  |  |  | 0.27 |
| BP treatment | 4144 (46.6%) | 1058 (47.6%) | 1060 (47.7%) | 1019 (45.8%) | 1007 (45.4%) |  |
| Lipid treatment | 4743 (53.4%) | 1166 (52.4%) | 1160 (52.3%) | 1205 (54.2%) | 1212 (54.6%) |  |
| Cigarette smoking |  |  |  |  |  | <0.001 |
| Yes | 1219 (13.7%) | 404 (18.2%) | 266 (12.0%) | 288 (12.9%) | 261 (11.8%) |  |
| No | 7668 (86.3%) | 1820 (81.8%) | 1954 (88.0%) | 1936 (87.1%) | 1958 (88.2%) |  |
| Systolic BP, mmHg | 135 (124, 146) | 135 (124, 146) | 135 (125, 146) | 135 (124, 146) | 135 (124, 146) | 0.68 ^Ψ^ |
| Diastolic BP, mmHg | 75 (68, 82) | 74 (66, 81) | 74 (68, 81) | 75 (68, 82) | 76 (69, 82) | <0.001 ^Ψ^ |
| CVD |  |  |  |  |  | 0.44 |
| Yes | 3087 (34.7%) | 759 (34.1%) | 790 (35.6%) | 790 (35.5%) | 748 (33.7%) |  |
| No | 5800 (65.3%) | 1465 (65.9%) | 1430 (64.4%) | 1434 (64.5%) | 1471 (66.3%) |  |
| Dyslipidemia |  |  |  |  |  | 0.002 |
| Yes | 1919 (21.6%) | 462 (20.8%) | 443 (20.0%) | 472 (21.2%) | 542 (24.4%) |  |
| No | 6968 (78.4%) | 1762 (79.2%) | 1777 (80.0%) | 1752 (78.8%) | 1677 (75.6%) |  |
| HDL, mg/dL | 40 (34, 48) | 41 (35, 50) | 40 (34, 47) | 39 (34, 47) | 40 (34, 47) | <0.001 ^Ψ^ |
| LDL, mg/dL | 101 (81, 125) | 102 (82, 127) | 99 (81, 123) | 101 (81, 125) | 100 (80, 124) | 0.12 ^Ψ^ |
| eGFR, ml/min/1.73m^2^ | 89.8 (76.3, 105.2) | 89.64999 (77.1, 104.8) | 89.4 (75.7, 104.7) | 89.8 (76, 105.3) | 90.2 (75.7, 106.3) | 0.26 ^Ψ^ |
| UACR, mg/g | 13 (7, 34) | 12 (6, 32.5) | 12 (6, 31) | 13 (7, 32) | 15 (7, 41) | <0.001 ^Ψ^ |
| Serum creatinine, mg/dL | .9 (.8, 1) | .9 (.8, 1) | .9 (.8, 1) | .9 (.7, 1) | .8 (.7, 1) | <0.001 ^Ψ^ |
| Abbreviations: WC= Waist circumference, WHtR= Waist-to-height ratio, BMI= Body mass index, BP= blood pressure, CVD= Cardiovascular disease, HDL= high density lipoprotein, LDL= Low density lipoprotein, eGFR= Estimated glomerular filtration rate, UACR= Urinary albumin creatinine ratio  Ψ: P-values from the rank sum test. All other p-values are from the Pearson chi-square test. | | | | | | |

**Supplementary Table 5** Characteristics of study participants according to BMI categories

| Characteristics | Total (n=8887) | Normal weight (n=727) | Overweight (n=2599) | Class I obese (n=2942) | Class II obese (n=1769) | Class III obese (n=850) | p-value |
| --- | --- | --- | --- | --- | --- | --- | --- |
| Age, yrs | 62.0 (57.7, 67.1) | 63.7 (58.3, 69.9) | 63.2 (58.6, 68.7) | 62.2 (57.8, 67.1) | 60.8 (57.2, 65.1) | 59.8 (56.7, 64.2) | <0.001 ^Ψ^ |
| Gender |  |  |  |  |  |  | <0.001 |
| Women | 3398 (38.2%) | 251 (34.5%) | 811 (31.2%) | 1069 (36.3%) | 784 (44.3%) | 483 (56.8%) |  |
| Men | 5489 (61.8%) | 476 (65.5%) | 1788 (68.8%) | 1873 (63.7%) | 985 (55.7%) | 367 (43.2%) |  |
| Race |  |  |  |  |  |  | <0.001 |
| White | 5596 (63.0%) | 311 (42.8%) | 1507 (58.0%) | 1954 (66.4%) | 1223 (69.1%) | 601 (70.7%) |  |
| Non-white | 3291 (37.0%) | 416 (57.2%) | 1092 (42.0%) | 988 (33.6%) | 546 (30.9%) | 249 (29.3%) |  |
| Education |  |  |  |  |  |  | <0.001 |
| Less than high school graduate | 1295 (14.6%) | 114 (15.7%) | 416 (16.0%) | 430 (14.6%) | 235 (13.3%) | 100 (11.8%) |  |
| High school grad (or GED) | 2342 (26.4%) | 186 (25.6%) | 679 (26.1%) | 781 (26.5%) | 483 (27.3%) | 213 (25.1%) |  |
| Some college or technical school | 2912 (32.8%) | 174 (24.0%) | 791 (30.4%) | 979 (33.3%) | 617 (34.9%) | 351 (41.3%) |  |
| College graduate or more | 2333 (26.3%) | 252 (34.7%) | 712 (27.4%) | 752 (25.6%) | 432 (24.4%) | 185 (21.8%) |  |
| WC, cm | 106.7 (97, 116) | 86.4 (81.3, 91.4) | 98 (92.7, 103) | 108 (102, 114) | 117 (111, 123.6) | 125.7 (118.5, 132.1) | <0.001 ^Ψ^ |
| Weight, kg | 92.6 (80.7, 105.7) | 67.1 (60.6, 73.5) | 81.6 (74.4, 88.9) | 95 (86.5, 102.9) | 107.7 (97.5, 117.5) | 118.8 (109.1, 127.9) | <0.001 ^Ψ^ |
| WHtR | .6 (.6, .7) | .5 (.5, .5) | .6 (.5, .6) | .6 (.6, .7) | .7 (.7, .7) | .8 (.7, .8) | <0.001 ^Ψ^ |
| BMI, kg/m^2^ | 31.8 (28.2, 35.8) | 23.7 (22.6, 24.4) | 27.9 (26.7, 29) | 32.3 (31.2, 33.6) | 37 (35.9, 38.3) | 42.1 (41, 43.4) | <0.001 ^Ψ^ |
| Hba1c, % | 8.1 (7.6, 8.8) | 8.1 (7.5, 8.9) | 8 (7.5, 8.8) | 8.1 (7.6, 8.8) | 8.1 (7.6, 8.8) | 8.1 (7.6, 8.9) | 0.034 ^Ψ^ |
| Duration of diabetes, yrs | 9 (5, 15) | 10 (5, 16) | 10 (5, 16) | 8 (5, 14) | 9 (5, 14) | 8 (5, 14) | <0.001 ^Ψ^ |
| Glycemia |  |  |  |  |  |  | 0.78 |
| Standard | 4438 (49.9%) | 348 (47.9%) | 1292 (49.7%) | 1478 (50.2%) | 887 (50.1%) | 433 (50.9%) |  |
| Intensive | 4449 (50.1%) | 379 (52.1%) | 1307 (50.3%) | 1464 (49.8%) | 882 (49.9%) | 417 (49.1%) |  |
| BP vs Lipid |  |  |  |  |  |  | 0.022 |
| BP treatment | 4144 (46.6%) | 369 (50.8%) | 1237 (47.6%) | 1318 (44.8%) | 809 (45.7%) | 411 (48.4%) |  |
| Lipid treatment | 4743 (53.4%) | 358 (49.2%) | 1362 (52.4%) | 1624 (55.2%) | 960 (54.3%) | 439 (51.6%) |  |
| Cigarette smoking |  |  |  |  |  |  | <0.001 |
| Yes | 1219 (13.7%) | 155 (21.3%) | 415 (16.0%) | 363 (12.3%) | 198 (11.2%) | 88 (10.4%) |  |
| No | 7668 (86.3%) | 572 (78.7%) | 2184 (84.0%) | 2579 (87.7%) | 1571 (88.8%) | 762 (89.6%) |  |
| Systolic BP, mmHg | 135 (124, 146) | 136 (124, 147) | 135 (125, 146) | 134 (124, 146) | 135 (124, 147) | 135 (124, 146) | 0.41 ^Ψ^ |
| Diastolic BP, mmHg | 75 (68, 82) | 73 (65, 80) | 73 (67, 80) | 75 (68, 82) | 76 (69, 82) | 77 (70, 83) | <0.001 ^Ψ^ |
| CVD |  |  |  |  |  |  | 0.023 |
| Yes | 3087 (34.7%) | 233 (32.0%) | 941 (36.2%) | 1055 (35.9%) | 587 (33.2%) | 271 (31.9%) |  |
| No | 5800 (65.3%) | 494 (68.0%) | 1658 (63.8%) | 1887 (64.1%) | 1182 (66.8%) | 579 (68.1%) |  |
| Dyslipidemia |  |  |  |  |  |  | <0.001 |
| Yes | 1919 (21.6%) | 154 (21.2%) | 497 (19.1%) | 627 (21.3%) | 434 (24.5%) | 207 (24.4%) |  |
| No | 6968 (78.4%) | 573 (78.8%) | 2102 (80.9%) | 2315 (78.7%) | 1335 (75.5%) | 643 (75.6%) |  |
| HDL, mg/dL | 40 (34, 48) | 44 (36, 53) | 40 (34, 48) | 39 (34, 46) | 39 (34, 47) | 40 (34, 48) | <0.001 ^Ψ^ |
| LDL, mg/dL | 101 (81, 125) | 104 (83, 128) | 100 (81, 124) | 100 (80, 124) | 100 (81, 124) | 102 (81, 128) | 0.18 ^Ψ^ |
| eGFR, ml/min/1.73m^2^ | 89.8 (76.3, 105.2) | 90 (77.1, 106) | 89.2 (76.1, 104.6) | 89.7 (76.2, 104.8) | 90.3 (76, 106.1) | 90.25 (76.4, 105.9) | 0.15 ^Ψ^ |
| UACR, mg/g | 13 (7, 34) | 13 (7, 36) | 13 (7, 32) | 12 (7, 32) | 14 (7, 37) | 13 (7, 43) | 0.001 ^Ψ^ |
| Serum creatinine, mg/dL | .9 (.8, 1) | .9 (.7, 1) | .9 (.8, 1) | .9 (.8, 1) | .9 (.7, 1) | .8 (.7, 1) | <0.001 ^Ψ^ |
| Abbreviations: WC= Waist circumference, WHtR= Waist-to-height ratio, BMI= Body mass index, BP= blood pressure, CVD= Cardiovascular disease, HDL= high density lipoprotein, LDL= Low density lipoprotein, eGFR= Estimated glomerular filtration rate, UACR= Urinary albumin creatinine ratio  Ψ: P-values from the rank sum test. All other p-values are from the Pearson chi-square test. | | | | | | | |

**Supplementary Table 6** Hazard ratios for SCr doubling or >20 mL/min decrease in eGFR, by WC, WHtR, and BMI among men with diabetes

|  | Events | Incidence Density (Per 10 000 Person Days) | Model 1 | Model 2 |
| --- | --- | --- | --- | --- |
| **WC group** |  |  |  |  |
| <102cm | 922 | 5.4 | Ref. | Ref. |
| ≥102cm | 2256 | 6.1 | 1.11 (1.03-1.20) * | 1.04 (0.96-1.12) |
|  |  |  |  |  |
|  |  |  |  |  |
| **WHtR Quartiles** |  |  |  |  |
| Q1 | 746 | 5.2 | Ref. | Ref. |
| Q2 | 776 | 5.8 | 1.08 (0.98-1.20) | 1.07 (0.97-1.18) |
| Q3 | 839 | 6.3 | 1.18 (1.07-1.30) ** | 1.13 (1.03-1.25) * |
| Q4 | 817 | 6.5 | 1.20 (1.08-1.32) *** | 1.06 (0.96-1.18) |
|  |  |  |  |  |
|  |  |  |  |  |
| **BMI categories** |  |  |  |  |
| Normal | 258 | 5.5 | Ref. | Ref. |
| Overweight | 1017 | 5.7 | 1.03 (0.90-1.18) | 1.02 (0.98-1.17) |
| Class I obese | 1106 | 6.1 | 1.08 (0.94-1.24) | 1.07 (0.93-1.23) |
| Class II obese | 567 | 5.8 | 1.05 (0.91-1.22) | 0.96 (0.83-1.12) |
| Class III obese | 230 | 7.1 | 1.22 (1.02-1.46) * | 1.08 (0.90-1.30) |
| Abbreviations: WC= Waist circumference; WHtR= Waist to height ratio; BMI= Body mass index Model 1 = unadjusted Model 2 adjusted for baseline age(cont.), sex (men or women), race (white or non-white), BP vs lipid (BP treatment and lipid treatment), duration of diabetes (cont.) systolic BP (cont.), HbA1c (cont.), eGFR (cont.), UACR (cont.), CVD history (yes or no), high-density lipoprotein (cont.) and low-density lipoprotein *P*-value notation: *: *P*<0.05. **: *P*<0.01. ***: *P*<0.001 | | | | |

**Supplementary Table 7** Hazard ratios for incident Macroalbuminuria, by WC, WHtR, and BMI among men with diabetes

|  | Events | Incidence Density (Per 10 000 Person Days) | Model 1 | Model 2 |
| --- | --- | --- | --- | --- |
|  |  |  |  |  |
| **WC group** |  |  |  |  |
| <102cm | 131 | 4.7 | Ref. | Ref. |
| ≥102cm | 274 | 4.3 | 0.93 (0.75-1.14) | 0.76 (0.62-0.95) * |
|  |  |  |  |  |
| **WHtR Quartiles** |  |  |  |  |
| Q1 | 105 | 4.5 | Ref. | Ref. |
| Q2 | 93 | 4.1 | 0.92 (0.69-1.21) | 0.79 (0.60-1.05) |
| Q3 | 95 | 4.1 | 0.92 (0.69-1.21) | 0.76 (0.57-1.01) |
| Q4 | 112 | 5.1 | 1.14 (0.88-1.49) | 0.82 (0.62-1.08) |
|  |  |  |  |  |
|  |  |  |  |  |
| **BMI categories** |  |  |  |  |
| Normal | 38 | 4.8 | Ref. | Ref. |
| Overweight | 134 | 4.4 | 0.92 (0.64-1.31) | 0.90 (0.62-1.29) |
| Class I obese | 130 | 4.2 | 0.87 (0.61-1.25) | 0.83 (0.57-1.20) |
| Class II obese | 67 | 4.1 | 0.85 (0.57-1.26) | 0.71 (0.47-1.07) |
| Class III obese | 36 | 6.2 | 1.32 (0.83-2.08) | 0.90 (0.56-1.43) |
| Abbreviations: WC= Waist circumference; WHtR= Waist to height ratio; BMI= Body mass index Model 1 = unadjusted Model 2 adjusted for baseline age(cont.), sex (men or women), race (white or non-white), BP vs lipid (BP treatment and lipid treatment), duration of diabetes (cont.) systolic BP (cont.), HbA1c (cont.), eGFR (cont.), UACR (cont.), CVD history (yes or no), high-density lipoprotein (cont.) and low-density lipoprotein *P*-value notation: *: *P*<0.05. **: *P*<0.01. ***: *P*<0.001 | | | | |

**Supplementary Table 8** Hazard ratios for renal failure or ESRD (dialysis) or serum creatinine (SCr) >3.3mg/dL in absence of an acute reversible cause, by WC, WHtR, and BMI among men with diabetes

|  | Events | Incidence Density (Per 100 000 Person Days) | Model 1 | Model 2 |
| --- | --- | --- | --- | --- |
| **WC group** |  |  |  |  |
| <102cm | 41 | 1.4 | Ref. | Ref. |
| ≥102cm | 103 | 1.6 | 1.12 (0.78-1.62) | 1.08 (0.74-1.55) |
|  |  |  |  |  |
|  |  |  |  |  |
| **WHtR Quartiles** |  |  |  |  |
| Q1 | 35 | 1.5 | Ref. | Ref. |
| Q2 | 43 | 1.9 | 1.26 (0.81-1.98) | 1.22 (0.78-1.93) |
| Q3 | 37 | 1.6 | 1.08 (0.68-1.71) | 1.04 (0.65-1.66) |
| Q4 | 29 | 1.3 | 0.88 (0.53-1.43) | 0.80 (0.49-1.32) |
|  |  |  |  |  |
|  |  |  |  |  |
| **BMI categories** |  |  |  |  |
| Normal | 14 | 1.7 | Ref. | Ref. |
| Overweight | 45 | 1.5 | 0.83 (0.46-1.51) | 0.80 (0.43-1.47) |
| Class I obese | 51 | 1.6 | 0.92 (0.51-1.66) | 0.85 (0.47-1.57) |
| Class II obese | 27 | 1.6 | 0.93 (0.49-1.77) | 0.81 (0.41-1.57) |
| Class III obese | 7 | 1.2 | 0.67 (0.27-1.66) | 0.56 (0.22-1.41) |
| Abbreviations: WC= Waist circumference; WHtR= Waist to height ratio; BMI= Body mass index Model 1 = unadjusted Model 2 adjusted for baseline age(cont.), sex (men or women), race (white or non-white), BP vs lipid (BP treatment and lipid treatment), duration of diabetes (cont.) systolic BP (cont.), HbA1c (cont.), eGFR (cont.), UACR (cont.), CVD history (yes or no), high-density lipoprotein (cont.) and low-density lipoprotein *P*-value notation: *: *P*<0.05. **: *P*<0.01. ***: *P*<0.001 | | | | |

**Supplementary Table 9** Hazard ratios for SCr doubling or >20 mL/min decrease in eGFR, by WC, WHtR, and BMI among women with diabetes

|  | Events | Incidence Density (Per 10 000 Person Days) | Model 1 | Model 2 |
| --- | --- | --- | --- | --- |
|  |  |  |  |  |
| **WC group** |  |  |  |  |
| <88cm | 195 | 4.5 | Ref. | Ref. |
| ≥88cm | 1656 | 5.6 | 1.20 (1.03-1.39) * | 1.39 (1.18-1.63) *** |
|  |  |  |  |  |
| **WHtR Quartiles** |  |  |  |  |
| Q1 | 445 | 5.0 | Ref. | Ref. |
| Q2 | 464 | 5.5 | 1.06 (0.93-1.21) | 1.14 (1.00-1.30) |
| Q3 | 457 | 5.5 | 1.07 (0.94-1.22) | 1.13 (0.99-1.29) |
| Q4 | 485 | 5.8 | 1.13 (0.99-1.29) | 1.15 (1.00-1.31) * |
|  |  |  |  |  |
|  |  |  |  |  |
| **BMI categories** |  |  |  |  |
| Normal | 123 | 4.6 | Ref. | Ref. |
| Overweight | 439 | 5.4 | 1.12 (0.91-1.36) | 1.40 (1.12-1.75) ** |
| Class I obese | 579 | 5.4 | 1.12 (0.93-1.37) | 1.43 (1.15-1.79) ** |
| Class II obese | 445 | 6.0 | 1.21 (0.99-1.48) | 1.49 (1.19-1.86) ** |
| Class III obese | 265 | 5.3 | 1.13 (0.91-1.39) | 1.40 (1.10-1.78) * |
|  |  |  |  |  |
| Abbreviations: WC= Waist circumference; WHtR= Waist to height ratio; BMI= Body mass index Model 1 = unadjusted Model 2 adjusted for baseline age(cont.), sex (men or women), race (white or non-white), BP vs lipid (BP treatment and lipid treatment), duration of diabetes (cont.) systolic BP (cont.), HbA1c (cont.), eGFR (cont.), UACR (cont.), CVD history (yes or no), high-density lipoprotein (cont.) and low-density lipoprotein *P*-value notation: *: *P*<0.05. **: *P*<0.01. ***: *P*<0.001 | | | | |

**Supplementary Table 10** Hazard ratios for incident Macroalbuminuria, by WC, WHtR, and BMI among women with diabetes

|  | Events | Incidence Density (Per 100 000 Person Days) | Model 1 | Model 2 |
| --- | --- | --- | --- | --- |
| **WC group** |  |  |  |  |
| <88cm | 20 | 2.9 | Ref. | Ref. |
| ≥88cm | 179 | 3.5 | 1.23 (0.78-1.96) | 0.98 (0.61-1.57) |
|  |  |  |  |  |
|  |  |  |  |  |
| **WHtR Quartiles** |  |  |  |  |
| Q1 | 42 | 2.9 | Ref. | Ref. |
| Q2 | 36 | 2.5 | 0.86 (0.55-1.34) | 0.91 (0.58-1.43) |
| Q3 | 56 | 4.0 | 1.40 (0.94-2.09) | 1.42 (0.94-2.14) |
| Q4 | 65 | 4.6 | 1.63 (1.11-2.41) * | 1.39 (0.93-2.07) |
|  |  |  |  |  |
|  |  |  |  |  |
| **BMI categories** |  |  |  |  |
| Normal | 15 | 3.5 | Ref. | Ref. |
| Overweight | 43 | 3.1 | 0.88 (0.49-1.59) | 0.69 (0.39-1.25) |
| Class I obese | 58 | 3.2 | 0.92 (0.52-1.63) | 0.89 (0.50-1.58) |
| Class II obese | 55 | 4.3 | 1.24 (0.70-2.19) | 1.03 (0.57-1.85) |
| Class III obese | 28 | 3.5 | 1.00 (0.53-1.87) | 0.79 (0.41-1.51) |
| Abbreviations: WC= Waist circumference; WHtR= Waist to height ratio; BMI= Body mass index Model 1 = unadjusted Model 2 adjusted for baseline age(cont.), sex (men or women), race (white or non-white), BP vs lipid (BP treatment and lipid treatment), duration of diabetes (cont.) systolic BP (cont.), HbA1c (cont.), eGFR (cont.), UACR (cont.), CVD history (yes or no), high-density lipoprotein (cont.) and low-density lipoprotein *P*-value notation: *: *P*<0.05. **: *P*<0.01. ***: *P*<0.001 | | | | |

**Supplementary Table 11** Hazard ratios for renal failure or ESRD (dialysis) or serum creatinine (SCr) >3.3mg/dL in absence of an acute reversible cause, by WC, WHtR, and BMI among women with diabetes

|  | Events | Incidence Density (Per 100 000 Person Days) | Model 1 | Model 2 |
| --- | --- | --- | --- | --- |
| **WC group** |  |  |  |  |
| <88cm | 7 | 1.0 | Ref. | Ref. |
| ≥88cm | 67 | 1.3 | 1.32 (0.60-2.87) | 1.32 (0.60-2.92) |
|  |  |  |  |  |
|  |  |  |  |  |
| **WHtR Quartiles** |  |  |  |  |
| Q1 | 13 | 0.9 | Ref. | Ref. |
| Q2 | 18 | 1.2 | 1.42 (0.69-2.89) | 1.40 (0.68-2.88) |
| Q3 | 18 | 1.2 | 1.43 (0.70-2.92) | 1.47 (0.71-3.03) |
| Q4 | 25 | 1.7 | 2.00 (1.03-3.92) * | 1.97 (0.99-3.94) |
|  |  |  |  |  |
|  |  |  |  |  |
| **BMI categories** |  |  |  |  |
| Normal | 3 | 0.7 | Ref. | Ref. |
| Overweight | 14 | 1.0 | 1.47 (0.42-5.12) | 1.59 (0.45-5.56) |
| Class I obese | 26 | 1.4 | 2.09 (0.63-6.91) | 2.24 (0.66-7.51) |
| Class II obese | 22 | 1.7 | 2.48 (0.74-8.30) | 2.62 (0.77-8.94) |
| Class III obese | 9 | 1.1 | 1.63 (0.44-6.03) | 1.77 (0.47-6.74) |
| Abbreviations: WC= Waist circumference; WHtR= Waist to height ratio; BMI= Body mass index Model 1 = unadjusted Model 2 adjusted for baseline age(cont.), sex (men or women), race (white or non-white), BP vs lipid (BP treatment and lipid treatment), duration of diabetes (cont.) systolic BP (cont.), HbA1c (cont.), eGFR (cont.), UACR (cont.), CVD history (yes or no), high-density lipoprotein (cont.) and low-density lipoprotein *P*-value notation: *: *P*<0.05. **: *P*<0.01. ***: *P*<0.001 | | | | |

**Supplementary Table 12** Subgroup analysis for association **o**f 1SD–increase in WC and risk of incident DN among men and women

|  | **Events/n** | **HR (95% CI)** | ***P* for interaction** |
| --- | --- | --- | --- |
| **Men** |  |  |  |
| **Age, yrs** |  |  |  |
| <65 | 2,154/3,511 | 1.00 (0.96 - 1.05) | 0.394 |
| ≥65 | 1,190/1,977 | 1.02 (0.96 - 1.09) |  |
| **Diabetes duration, yrs** |  |  |  |
| <9 | 1,551/2,602 | 1.04 (0.99 - 1.09) | 0.237 |
| ≥9 | 1,793/2,886 | 0.98 (0.94 - 1.03) |  |
| **BP vs lipid group** |  |  |  |
| BP treatment | 1,183/2,166 | 0.98 (0.92 - 1.05) | 0.369 |
| Lipid treatment | 2,161/3,322 | 1.02 (0.97 - 1.06) |  |
| **Systolic BP, mmHg** |  |  |  |
| <140 | 2,044/3,489 | 1.01 (0.97 - 1.06) | 0.594 |
| ≥140 | 1,300/1,999 | 1.00 (0.94 - 1.06) |  |
| **BMI, kg/m^2^** |  |  |  |
| ≤30 | 1,331/2,244 | 1.03 (0.94 - 1.13) | 0.873 |
| >30 | 2,013/3,244 | 1.00 (0.95 - 1.06) |  |
|  |  |  |  |
| **Women** |  |  |  |
| **Age, yrs** |  |  |  |
| <65 | 1,384/2,345 | 1.06 (1.00 - 1.12) * | 0.382 |
| ≥65 | 567/1,052 | 1.06 (0.97 - 1.16) |  |
| **Diabetes duration, yrs** |  |  |  |
| <9 | 894/1,584 | 1.11 (1.03 - 1.19) ** | 0.064 |
| ≥9 | 1,057/1,813 | 1.04 (0.97 - 1.11) |  |
| **BP vs lipid group** |  |  |  |
| BP treatment | 1,089/1,977 | 1.10 (1.03 - 1.17) ** | 0.044 |
| Lipid treatment | 862/1,420 | 1.01 (0.94 - 1.09) |  |
| **Systolic BP, mmHg** |  |  |  |
| <140 | 1,096/2,029 | 1.06 (0.99 - 1.12) | 0.325 |
| ≥140 | 855/1,368 | 1.09 (1.01 - 1.17) * |  |
| **BMI, kg/m^2^** |  |  |  |
| ≤30 | 583/1,057 | 1.07 (0.94 - 1.22) | 0.178 |
| >30 | 1,368/2,340 | 1.02 (0.96 - 1.10) |  |
| Each stratification was adjusted for baseline age, sex, race, BP vs lipid, duration of diabetes, systolic BP, HbA1c, eGFR, UACR, CVD history, high-density lipoprotein and low-density lipoprotein except for the stratification factor itself. Note: CI = confidence interval, BP = blood pressure, HR = hazard ratio, WC = waist circumference, BMI = body mass index *p*-value notation: *: p<0.05. **: p<0.01. ***: p<0.001 | | | |

**Supplementary Table 13** Subgroup analysis for association **o**f 1SD–increase in WHtR and risk of incident DN among men and women

|  | **Events/n** | **HR (95% CI)** | ***P* for interaction** |
| --- | --- | --- | --- |
| **Men** |  |  |  |
| **Age, yrs** |  |  |  |
| <65 | 2,154/3,511 | 1.01 (0.97 - 1.06) | 0.425 |
| ≥65 | 1,190/1,977 | 1.03 (0.97 - 1.10) |  |
| **Diabetes duration, yrs** |  |  |  |
| <9 | 1,551/2,602 | 1.03 (0.98 - 1.08) | 0.521 |
| ≥9 | 1,793/2,886 | 1.00 (0.96 - 1.05) |  |
| **BP vs lipid group** |  |  |  |
| BP treatment | 1,183/2,166 | 0.99 (0.93 - 1.05) | 0.333 |
| Lipid treatment | 2,161/3,322 | 1.03 (0.98 - 1.07) |  |
| **Systolic BP, mmHg** |  |  |  |
| <140 | 2,044/3,489 | 1.02 (0.98 - 1.07) | 0.401 |
| ≥140 | 1,300/1,999 | 1.00 (0.94 - 1.06) |  |
| **BMI, kg/m^2^** |  |  |  |
| <30 | 1,331/2,244 | 1.05 (0.96 - 1.15) | 0.841 |
| ≥30 | 2,013/3,244 | 1.01 (0.96 - 1.07) |  |
|  |  |  |  |
| **Women** |  |  |  |
| **Age, yrs** |  |  |  |
| <65 | 1,384/2,345 | 1.06 (1.01 - 1.12) * | 0.389 |
| ≥65 | 567/1,052 | 1.05 (0.97 - 1.15) |  |
| **Diabetes duration, yrs** |  |  |  |
| <9 | 894/1,584 | 1.09 (1.02 - 1.17) * | 0.206 |
| ≥9 | 1,057/1,813 | 1.05 (0.99 - 1.12) |  |
| **BP vs lipid group** |  |  |  |
| BP treatment | 1,089/1,977 | 1.10 (1.04 - 1.17) ** | 0.028 |
| Lipid treatment | 862/1,420 | 1.01 (0.94 - 1.08) |  |
| **Systolic BP, mmHg** |  |  |  |
| <140 | 1,096/2,029 | 1.05 (0.99 - 1.12) | 0.424 |
| ≥140 | 855/1,368 | 1.08 (1.01 - 1.16) * |  |
| **BMI, kg/m^2^** |  |  |  |
| ≤30 | 583/1,057 | 1.07 (0.94 - 1.21) | 0.178 |
| >30 | 1,368/2,340 | 1.02 (0.96 - 1.10) |  |
| Each stratification was adjusted for baseline age, sex, race, BP vs lipid, duration of diabetes, systolic BP, HbA1c, eGFR, UACR, CVD history, high-density lipoprotein and low-density lipoprotein except for the stratification factor itself. Note: CI = confidence interval, BP = blood pressure, HR = hazard ratio, WC = waist circumference, BMI = body mass index *p*-value notation: *: p<0.05. **: p<0.01. ***: p<0.001 | | | |

**Supplementary Table 14** Subgroup analysis for association **o**f 1SD–increase in BMI and risk of incident DN among men and women

|  | **Events/n** | **HR (95% CI)** | ***P* for interaction** |
| --- | --- | --- | --- |
| **Men** |  |  |  |
| **Age, yrs** |  |  |  |
| <65 | 2,154/3,511 | 0.99 (0.95 - 1.03) | 0.245 |
| ≥65 | 1,190/1,977 | 1.02 (0.96 - 1.09) |  |
| **Diabetes duration, yrs** |  |  |  |
| <9 | 1,551/2,602 | 1.04 (0.99 - 1.09) | 0.664 |
| ≥9 | 1,793/2,886 | 0.98 (0.94 - 1.03) |  |
| **BP vs lipid group** |  |  |  |
| BP treatment | 1,183/2,166 | 0.99 (0.93 – 1.05) | 0.590 |
| Lipid treatment | 2,161/3,322 | 1.01 (0.97 - 1.05) |  |
| **Systolic BP, mmHg** |  |  |  |
| <140 | 2,044/3,489 | 1.01 (0.97 - 1.06) | 0.593 |
| ≥140 | 1,300/1,999 | 0.99 (0.94 - 1.05) |  |
| **WC, cm** |  |  |  |
| <105 | 1,352/2,268 | 1.02 (0.93 - 1.11) | 0.811 |
| ≥105 | 1,992/3,220 | 1.03 (0.97 - 1.09) |  |
|  |  |  |  |
| **Women** |  |  |  |
| **Age, yrs** |  |  |  |
| <65 | 1,384/2,345 | 1.04 (0.98 - 1.09) | 0.896 |
| ≥65 | 567/1,052 | 1.08 (0.99 - 1.18) |  |
| **Diabetes duration, yrs** |  |  |  |
| <9 | 894/1,584 | 1.11 (1.03 - 1.19) ** | 0.023 |
| ≥9 | 1,057/1,813 | 1.04 (0.97 - 1.11) |  |
| **BP vs lipid group** |  |  |  |
| BP treatment | 1,089/1,977 | 1.09 (1.02 - 1.15) * | 0.026 |
| Lipid treatment | 862/1,420 | 1.00 (0.93 - 1.08) |  |
| **Systolic BP, mmHg** |  |  |  |
| <140 | 1,096/2,029 | 1.04 (0.98 - 1.10) | 0.434 |
| ≥140 | 855/1,368 | 1.07 (0.99 - 1.14) |  |
| **WC, cm** |  |  |  |
| <105 | 987/1,773 | 1.12 (1.02 - 1.23) * | 0.003 |
| ≥105 | 964/1,624 | 0.96 (0.88 - 1.05) |  |
| Each stratification was adjusted for baseline age, sex, race, BP vs lipid, duration of diabetes, systolic BP, HbA1c, eGFR, UACR, CVD history, high-density lipoprotein and low-density lipoprotein except for the stratification factor itself. Note: CI = confidence interval, BP = blood pressure, HR = hazard ratio, WC = waist circumference, BMI = body mass index *p*-value notation: *: p<0.05. **: p<0.01. ***: p<0.001 | | | |

**Supplementary Table 15** Sensitivity analysis of WC, WHtR and BMI in relation to DN by excluding serious adverse event occurring early in the first two years of follow-up among men

|  |  | **HR (95% CI)** |
| --- | --- | --- |
| **WC, cm** |  |  |
| **<**102 |  | Ref. |
| **≥**102 |  | 1.03 (0.95 – 1.11) |
|  |  |  |
| Each SD increase |  | 1.01 (0.98 – 1.05) |
|  |  |  |
| **Quartiles WHtR** |  |  |
| 1 |  | Ref. |
| 2 |  | 1.07 (0.97 – 1.18) |
| 3 |  | 1.11 (1.01 – 1.23) * |
| 4 |  | 1.07 (0.97 – 1.18) |
|  |  |  |
| Each SD increase |  | 1.02 (0.99 – 1.06) |
|  |  |  |
| **BMI group** |  |  |
| Normal |  | Ref. |
| Overweight |  | 1.05 (0.91 – 1.20) |
| Class 1 obese |  | 1.09 (0.95 – 1.25) |
| Class 2 obese |  | 0.98 (0.84 – 1.14) |
| Class 3 obese |  | 1.15 (0.96 – 1.37) |
|  |  |  |
| Each SD increase in BMI |  | 1.01 (0.97 – 1.05) |

Model was adjusted for baseline age, sex, race, BP vs lipid, duration of diabetes, systolic BP, HbA1c, eGFR, UACR, CVD history, high-density lipoprotein and low-density lipoprotein

Note: CI = confidence interval, DN = diabetic nephropathy, WC = waist circumference, WHtR = waist to height ratio, BMI = body mass index, HR = hazard ratio, SD = standard deviation.

P-value notation: *: p<0.05. **: p<0.01. ***: p<0.001

**Supplementary Table 16** Sensitivity analysis of WC, WHtR and BMI in relation to DN by excluding serious adverse event occurring early in the first two years of follow-up among women

|  |  | **HR (95% CI)** |
| --- | --- | --- |
| **WC, cm** |  |  |
| **<**88 |  | Ref. |
| **≥**88 |  | 1.33 (1.14 – 1.55) *** |
|  |  |  |
| Each SD increase |  | 1.07 (1.02 – 1.12) * |
|  |  |  |
| **Quartiles WHtR** |  |  |
| 1 |  | Ref. |
| 2 |  | 1.08 (0.95 – 1.23) |
| 3 |  | 1.11 (0.97 – 1.26) |
| 4 |  | 1.13 (0.98 – 1.29) |
|  |  |  |
| Each SD increase |  | 1.07 (1.02 – 1.12) * |
|  |  |  |
| **BMI group** |  |  |
| Normal |  | Ref. |
| Overweight |  | 1.25 (1.02 – 1.55) * |
| Class 1 obese |  | 1.34 (1.09 – 1.64) * |
| Class 2 obese |  | 1.40 (1.13 – 1.73) ** |
| Class 3 obese |  | 1.31 (1.04 – 1.65) * |
|  |  |  |
| Each SD increase in BMI |  | 1.05 (1.00 – 1.10) * |

Model was adjusted for baseline age, sex, race, BP vs lipid, duration of diabetes, systolic BP, HbA1c, eGFR, UACR, CVD history, high-density lipoprotein and low-density lipoprotein

Note: CI = confidence interval, DN = diabetic nephropathy, WC = waist circumference, WHtR = waist to height ratio, BMI = body mass index, HR = hazard ratio, SD = standard deviation.

P-value notation: *: p<0.05. **: p<0.01. ***: p<0.001
